# Intermittent Fever—Its Various Forms—Their Treatment

**Published:** 1847-04

**Authors:** Lewis D. Ford

**Affiliations:** Professor of the Institutes and Practice of Medicine in the Medical College of Georgia


### PART IV.—SELECTIONS.

1. *Intermittent Fever — Its various forms — their treatment — abortive treatment of Remittent Fever.* By Lewis D. Ford, M. D., Professor of the Institutes and Practice of Medicine in the Medical College of Georgia.

In continuation of this subject, commenced in No. 9, Vol. 1, of this Journal, the writer passes to that form commonly called Remittent fever.

If, as has been shown, it is the duty and interest of the practictioner of this climate to understand the lineaments and the pathology of malignant Intermittent fever; how much greater is the obligation upon him to know well the nature of Remittent — the former being of comparatively rare occurrence, whilst the latter may be called, emphatically, *the disease* of the Southern clime, constituting, as it does, the great mass of his cases, in the summer and autumn; and the result of his treatment of this fever determining his professional reputation.

The popular, almost universal name of Bilious fever it may be remarked, in passing, is highly objectionable — a name suggested by the marked disorders of the biliary secretions usually present, and by the pathology which regards it as dependent essentially, upon disease of the liver. Manifestly inappropriate to those cases not characterized by bilious disorder, it is, at best, an unfortunate name, because of the prejudice it creates in weak minds, that vitiated bile or a diseased state of the liver is its proximate cause. There is a peculiar propriety in the simple name of Remittent; this, describing the prominent characteristic feature of its every variety, and directing the mind to its *paroxysmal* character as the most essontial feature, and giving a bias to enquiry into the nature of *paroxysmal* fevers, whichdirects to the knowledge of their nature.

Remittent fevers are characterized by an abatement of all their symptoms, at regular periods, generally once in twenty-four hours. This state of remission differs from an intermission, in that there are still present the febrile symptoms, but these diminished in violence. Individual cases, having this one feature of remittency in common, yet vary in their other symptoms, and in the organ or organs prominently affected; so that it is impossible to give a general description of remittent fever, which shall embrace all its varieties. It is therefore proposed to notice very generally the simplest form, and then its most difficult and dangerous varieties. The object of the writer being rather to develope the pathology and treatment, it will not be necessary to give at large, the symptoms of simple remittent fever, and he therefore refers to the description of this to be found in systematic writers. The remark upon reading these descriptions is obvious, that they do but detail the symptoms of a paroxysm of Intermittent. Cullen, for example, embraces both forms under the one head of intermittents, alluding to the difference in the duration and degree of the intermission, as the only difference in their external characters. And, when described by others as different forms, yet it is but the re-production of the symptoms of the intermittent form. So that it may be considered a fact, that cases of *simple* remittent fever do occur in this climate, and this is generally in the early summer, and run their course, for many days, without any more prominent local affection than in intermittents, and often terminate in intermittents.

In referring to the graver forms of this fever up to the most malignant, it may be remarked, that all these are marked by some predominating affection of one or more vital organs, thus giving individuality to each. Thus, a common form is that, which may be called cephalitic remittent—in which there is violent pain in the head, and giddiness and intolerance of low degrees of light and sound; these symptoms alternating with high maniacal delirium, and accompanied with nausea and even vomiting—these latter symptoms, evidently, not dependent on the grastritic state, but having the same relation to the state of the brain as they have in idiophathic phrenitis, uncomplicated with gastritris—sympathetic gastric disturbances, capable of being calmed by antiemetics. If unrelieved, this form has a rapid course, and terminates with the symptoms of the last stage of fatal phrenitis.

A still more common form is that marked by head-ache and even delirium; but these are not as prominent and distressing symptoms as persevering nausea and violent uncontrolable vomiting with a red dry tongue, or furred yellow, or brown in the middle its edges and end only red and dry, with pain at the epigastrium increased by pressure, the bowels generally loose—constituting gastric remittent. Another is enteritic remittent, characterized by diarrhœa, which is notably increased at every succeeding paroxysm, and by even mild laxatives. Each one of these forms is pretty uniformly accompanied with disorders of the biliary secretions. But there are other forms, characterized chiefly by these bilious derangements; as by an excessive secretion of bile of a healthy color, a bright yellow color. poured out in such quantity as to regurgitate into the stomach and produce nausea and vomiting of this bile, and by bilious purging. Another variety in the biliary secretion is the entirely opposite of this, dependent on a more serious and more controlling disorder of the liver—a suspended secretion, accompanied with nausea and vomiting, but no bile discharged. With fulness, heaviness, and oppression of the epigastrium, sighing and general restlessness, a dull head-ache, dingy color of the skin, torpid bowels, which under the operation of saline and even drastic cathartics, do not discharge bilious stools.

Each one of these forms of remittent, may terminate in the typhoid state, with its characteristic symptoms of delirium, *subsultus tendinum*, extremely frequent pulse, diarrhœa and tympanitis.

The most fatal, or most rapidly fatal, is the algid form—characterized by imperfect reaction, unequal distribution of animal heat, cold extremities, and coldness of the general surface, and disordered sensation—a sense of heat at the surface as well as internal heat, with oppression of the chest and epigastrium, laborious respiration, jactitaion, &c. This old form is now better known under the new name of *Congestive Fever*.

In reviewing the opinions of the profession on the pathology of remittents, it strikes the writer that too much importance has been given to the local congestions and inflammations, which form universally a part and parcel of the more serious cases of the disease—that too much reliance has been placed upon *post-mortem* appearances, as indicative of original and substantial disease—that the accidents and consequences of of the disease have been mistaken for its original basis. Allowing for a moment that the evidences of gastro-intestinal inflammation were much more frequent than observation determines them to be—that they were found in every case; the conclusion is by no means warranted, that the disease is substantially a gastro-enteritis; it is as absurd as would be the conclusion that gastro intestinal inflammation formed no part of infantile remittent fever, merely because autopsic examination found the physical traces of disease in the cavity of the cranium, and the stomach and intestines sound. Whilst the information of pathological anatomy in this disease has a great value, yet the interpretation of the functional symptoms is more to be relied upon, in determining the location, at least, of its primitive irritation.

However the forms of remittent fever may be varied by the predominance of local symptoms, this character of periodicity marks them all—the disease is equally *paroxysmal*, when characterized by encephalitic symptoms as by gastric—*paroxysmal* in the thoracic varieties, and still *paroxysmal* in the simple form, which is characterized by no more prominent local disease than exists in the paroxysm of the simplest intermittent; and therefore this periodicity cannot be dependent upon any one of these local affections, it must depend upon some affection of some part of the system, as uniformly present as this remittence. These local affections, then, how violent soever they may be—how controlling soever their influence upon the progress and final termination of remitting fever, may, with great propriety, be called complications or accidents, in reference to the fever itself. As to the relation of these complications to periodical fever, the writer referring to the cases adduced in the previous No. of this Journal, to prove the independence of the fever upon them, would remark further, that these accidents are manifestly not immaterial, but on the contrary, exert the most decided control over the regularity of the paroxysms, and are the immediate causes of their fatality. Thus, simple remittents are most regular in their paroxysms, and preserve this regularity throughout their whole course—the paroxysms more distinctly separated from each other; and, again, as the local affection becomes more fixed and more violent, in the same degree is the regularity of the paroxysms interrupted, the remissions are shorter and more obscure, until finally, with the complete establishment of the phlegmasial state upon the organ, the remissions cease and the case passes to the continued form. Thus it may be perceived that so far from phlegmasial disease determining the remittent fever, the very opposite is true—it destroys the type.

It is true of malignant remittent as of intermittent fevers, that they preserve their character of mild remittents, for some paroxysms, and gradually pass into the malignant; and of these the remark is universally true, that the local phlegmasia or congestion is increased by each succeeding paroxysm—that while the paroxysms are completed within its natural period of twenty-four hours, the local symptoms increase and abate with the increase and abatement of the paroxysm. To adopt the beautiful simile of Torti—"these wait upon the paroxysm, like the shadow upon the substance." So true is this, that in the vast majority of cases, when the paroxysm is broken up, the local affection subsides, without the necessity of addressing remedies to it—just what might reasonably be expected from observing its dependence upon the paroxysm.

That the local congestion or inflammation has no influence in determining the periodicity of remittent fever, will be manifest from the fact, that cases of mild remitttent fever do sometimes run their whole course during seven or ten days, without any local phlegmasia or congestion greater than is found in simple intermittents; that such cases used to be treated in former years, among us at least, greatly to the comfort of patients, by small repeated doses of tartar emetic—a medicine by the common consent of the profession, proscribed wherever there is the remotest suspicion of the existence of gastritis; to which latter affection it has been so fashionable of late, to refer as the primitive irritation in remittent fever.

If the remittent fever be independent of local congestions or inflammations, the proper cases to select for illustrating its pathology are the *simplest cases*, those uncomplicated by any adventitious accidents.

The characters of simple remittent fever show it to be essentially an intermittent. The simultaneous occurrence of intermittents and remittents, in the same locality—nay, in different members of the same household, all under common circumstances of living and of exposure, proves satisfactorily their dependence upon one and the same common cause. The symptoms of the paroxysm of simple remittent and intermittent fevers are so similar, that the most penetrating observer cannot with confidence, determine, during the passage of a first paroxysm, whether the case will develope itself as an intermittent or remittent. Again, what more common than the change of type from intermittent to remittent and *vice versa.* And the appeal is fearlessly made to practitioners—Is it not common to meet with paroxysmal fever, beginning as intermittents, continuing as remittents, and ending fatally by the supervention of a paroxysm marked by symptoms of the utmost malignancy?

As in intermittents so in remittents, one of the most uniform symptoms is tenderness to pressure in some portion or portions of the spinal chord; and, further, the controlling influence of spinal disease over the symptoms manifested in the head and in the various abdomininal viscera and over the muscular disorders, is illustrated by the efficacy of revulsives to the spinal column, in relieving all these distressing symptoms of the paroxysm.

The essential identity of intermittent and remittent fever is shown from another character of the latter form, alluded to by all practical writers, viz: its tendency to increase in the violence of its symptoms, on alternate days—at the tertian period. In fine, an inspection of the character of simple remittent fever shows no more difference between it and a quotidian intermittent, than between a quotidian and a tertian intermittent—a difference merely in the interval—that they are all essentially the same disease.

The conclusion to which the writer arrives as to the pathology of remittent fever in all its varieties, from the simplest to the most malignant, is, that remittent fever is an intermittent, rendered irregular by some complicating accident—that these complications, such as congestion and inflammation of one or more of the vital organs, so far from determining the remittency, tend to destroy it—that when produced by the paroxysm or by some peculiarity in the organs, they are increased by every succeeding paroxysm—that such is their dependence upon the paroxysm, that when this is checked these accidents disappear, without requiring subsequent local treatment; and that the fundamental lesion upon which depends remittent fever, is in the nervous centres—the spinal marrow and the brain.

This view of the nature of remittent fever indicates the same grand object to be accomplished in the treatment of each of its varied forms, viz: to prevent the recurrence of the paroxysms and to moderate the violence of their symptoms when present. Thus, as intermittents, the treatment naturally is divided into two parts—the one appropriate to the remission, the other to the exacerbation. Now, if an enlightened experience will sustain the course of practice, which this pathology indicates, it will add confirmation to its truth. To this test at last, must every system of treatment admit itself; for the writer is ready to acknowledge, in the face of all that has been written against the empiric method, that the so-called rational method rests upon empiricism—that all we know of the operation of medicines and of remedial methods is the result only of experience.

1. *To prevent the return of the paroxysms.*—This distinct indication to be accomplished in the treatment of *remittent fever* of recent origin, constrasts strikingly with the objects set forth in almost every system of practice, with which the writer is acquainted. In the light of an experience of twelve years' faithful adherence to this object, it is lamentable to look back upon his own previous practice and that of the whole body of medical men, directed according to the teachings of the many popular "Practices of Physic," these founded manifestly upon the notion, that remittent fever once fairly commenced, cannot be arrested in its course—teaching that symptoms are to be palliated as they arise, the fever all the while being permitted to renew its paroxysms, with all their increasing and fatal concomitants. The writer, conscious that he will be doing a service to his brother practitioners, whose attention may not as yet have been directed to this important point, turns to some of the most popular and recent of these hand-books, to substantiate his declaration.

Look, e. g., at the objects proposed in the treatment, in Eberle's Practice—a work which has had so large a share in forming the opinions of medical men and shaping their practice: "In the treatment of this disease, there are three primary pathological conditions, according to which the general indications of remediate management must be directed, viz: 1. Functional derangement of the liver and alimentary canal. 2. Redundancy of morbid or vitiated secretions, and consequent irritation in the intestinal tube. 3. An irritated increased action of the heart and arteries. Hence, the principal indications in the treatment are: 1, to moderate the febrile reaction of the arterial system; 2, to remove out of the alimentary canal, the vitiated and irritating secretions which may be lodged in it; 3, to restore the healthy functions of the alimentary canal; and 4, to obviate gastro-intestinal irritation."—Among the methods of treatment not a word is said of an effort to arrest it.

In Dunglison's practice, the whole routine system of bleeding, purging, sweating, refrigerating, blistering, &c., is examined, but not a word as to the abortive treatment.

The writer turns to the treatment of remittent fever in a work published in 1847, by Dr. Clymer, whose aim has been, "to adapt it particularly to the necessities of the American Practitioner," and reads—"The indications of treatment in remittent fever do not materially differ from those of continued fever. The points more particularly to be attended to, are the reduction of the general fever, obviating the effects of congestion and inflammatory action in the liver," and other organs. In a note, we are informed, that the simple expectant plan, is the one, which has been generally of late recommended by the experienced? At the end of the note the indication is stated, in the Congestive fever, to prevent the recurrence of the paroxysm.

In Watson's Practice by Condie, remittent fever forms the subject of a note—in which it is announced that the most important question that presents itself in the treatment is the propriety of direct depletion by the lancet! And in Professor Dickson's Lectures, commended especially to the southern student and practitioner, there is the same minute remarks upon blood-letting, emetics, cathartics, calomel, cold, &c., &c., but not one word upon what must be regarded as the leading rational object—the checking of the paroxysm. Indeed upon this point, the necessary continuance of the disease when once formed is distinctly, though incidentally asserted. "Could we reasonbly hope to prostrate the disease by a single blow, as is often done in the cure of phlegmasia, in pleurisy, &c., we might more implicitly trust to the lancet; *but the case is far otherwise.* Here the atmospheric and climatic predispositions are permanent, and the poisonous cause is still diffused around the patient, impressing the tissues with a continuous and UNAVOIDABLE agency. Success does not depend upon, nor can we hope or expect to attain it, by any single measure, however judicious and energetic."

In Professor Chapman's Syllabus, by Kennedy, published in 1846, quinine, the specific remedy for jugulating remittent fever, is classed among the *adjuvants* of the old routine system of practice.

And in Bell and Stokes' Practice, even in the latest edition, although the efficacy of the quinine practice is fully shown—the early unconditional use of quinine plainly set forth and triumphantly vindicated, yet in the treatment of the milder forms of remittent, this cardinal object of checking the recurrence of the paroxysm is not even hinted at. The writer, however, in passing, would pay the tribute of his high respect to the author of the articles on paroxysmal fevers, in this work; and express his sense of the obligations of the profession and of society to that author, for the general diffusion of modes of treatment, so admirably calculated to check the mortality of that hitherto fatal and always dangerous disease, congestive intermittent and remittent fever.

But where the propriety of confining the use of quinine to congestive remittent fever?—where the propriety of allowing simple remittent to run its course unchecked, whilst we hold in our hands a remedy so safe, so gentle, so certain as the sulphate of quinine? If it has the power of arresting the paroxysms of *malignant* remittent, in which, on the remittent fever is superadded the disturbing influences of extensive congestions and local inflammations, surely it must be able to control and arrest the *simple* form; and if so, there can be no propriety in allowing it to run its course unchecked; for who, that has lived where remittents are endemic, does not know, that a malignant paroxysm often supervenes, after many paroxysms of a mild and simple character; and that this paroxysm is dangerous in proportion to the previous duration of the fever: and, further, that simple remittent often lapses off into the typhoid state, to the imminent danger of the patient. Why run the hazard of these dangers by allowing its continuance?

*To prevent the recurrence of the paroxysms—to jugulate the disease.*—An analysis of the symptoms points to this then, as the prominent object, in every stage and every degree of the disease, as long, at least, as it preserves a paroxysmal character. Whilst it generally happens, that opportunity is afforded for the use of depletion, by bloodletting and other evacuations, during the paroxysm, yet the pathology which teaches that the remittent fever is the main affection, forbids us to allow the first remission to pass without attempting to accomplish this primary indication, if evacuations may not have been previously employed. This object may be accomplished by the use of sulphate of quinine—universally acknowledged to be the specific of intermittent fever, indicated also, as the specific of remittent, by the fundamental similarity of these two affections, and known to be so, by all who have thus used it. The interval between the paroxysms being shorter than in the intermittent form, the doses must necessarily be larger, in order to administer the requisite quantity, before the period of the next accession—from five to ten grains, hourly, according to the length of the remission, to the extent of fifteen, twenty, or fifty grains. For in determining the quantity, the rule laid down in the treatment of malignant intermittents, serves for a guide here, viz: the quantity to be directly proportioned to the degree of danger apprehended from the coming paroxysm; thus, in malignant remittents, the largest, and in simple remittents, the smallest quantity.

The writer must be content with stating the result of his own experience, in this mode of treatment: that generally it checks the first paroxysm, almost universally the second, in the milder forms of the disease—that the average time of attendance upon such cases is three or four days—that, when the quinine fails to arrest a coming paroxysm, it mitigates its violence, shortens its duration ; and although in some rare cases, the nervous symptoms produced by the remedy, are distressing to the patient during the paroxysm, these are soon relieved by the treatment appropriate to this state—that he has almost forgotten the features of a typhoid state of fever, so painfully familiar to him, previous to the last twelve years, when using the treatment then generally taught by authority and sanctioned by the profession.

Of this result the writer would say—those who have not fairly tried this mode of practice, have no right to question the justness of his conclusion—those who have, he confidently believes, will confirm it.

The writer does not undervalue the minute estimation of the circumstances, under which bloodletting, emetics, cathartics, mercurials and other remedies, should and should not be used, which is to be found in all the works on Practice; yet he declares his conviction, that the practitioner, holding steadily to this prominent indication, will find little need of availing himself of such instruction—that in the great majority of cases of simple Intermittent fever, by the use, during the paroxysm of bloodletting or not, and of the safest and surest emetic, water, (cold or warm, according to circumstances,) ingested into the stomach in such liberal quantities as to produce detergent vomiting, and this followed by a large injection to evacuate the bowels, and sinapisms to the vertebral column; the comfort of the patient is better secured, than by the administration of much physic, until the time arrives for the administration of the specific. If, after the subduction of the fever, there remains the evidence of disease in the liver, stomach or bowels, then this may be corrected by appropriate remedies, more readily, more safely and effectually than during the fever. The writer would insist upon this subsequent treatment of any remaining disease, as a necessary part of this abortive treatment.

How totally different the treatment here recommended for incipient remittent fever, from that in recently published books of Practice, may appear by the following quotation from Professor Dickson's issued as late as 1845:—"During the remission which the management above detailed as requisite throughout the course and progress of the exacerbation is intended to hasten, to render more perfect, and to prolong, you must not allow your attention to your patient to slacken. Nay, you are now called upon, perhaps, for a still nicer and more assiduous exertion of diligence and skill, as the improved circumstances often afford a better opportunity of useful interference. Purgatives, if formerly rejected, will now probably remain upon the stomach and act kindly. Diaphoretics, too are less apt to nauseate, and may be exhibited in fuller doses, and procure a more free and diffused sweating. It is thus that you may hope to diminish the violence of the returning exacerbation, if you cannot altogether prevent it. To subtract as much as possible from its intensity, time the administration of your prescriptions so as to bring your patient most completely under their effect, freely operated on by your purgative, fully sweated by your sudorific, just at the period of its expected invasion. Let his windows then be darkened, his apartment kept fresh and cool by ventilation, and, if necessary by evaporation, sprinkling his floor with water, vinegar, or ardent spirits, and prevent any excitement by noise or by conversation with him. It is advisable farther, to meet a coming exacerbation with revulsives so applied as to counteract or diminish the local determination to important organs." The writer declares his greater confidence in the silent operation of fifteen or twenty grains of quinine, during the remission, in the absence of the physician, than in the strictest *surveillance* of a whole college faculty, armed with their Cathartics, Sudorifics and Mustard-plasters.

The value of this treatment, if it be as successful as herein declared, will be the more highly appreciated, if we consider, at one view, the various terminations of remittent fever of the milder kind—that the most favorable is in convalescence at the end of a week or ten days, after the patient shall have undergone, not only all the anguish of fever, but in addition thereto, the annoyance of emetics, cathartics, nauseous sudorific draughts, ptyalism, perhaps flaying with vesicatories, and moreover, agitated, day after day, patients and friends, by the uncertainty of the final result—that another termination is the unexpected development of a malignant paroxysm, almost uniformly fatal, certainly so, with the continuance of the treatment which permitted it—that another is, the gradual loss of the remitting character and the establishment of the typhoid state, not as uniformly fatal, but imminently dangerous. The abortive treatment secures an early convalescence, saving the patient many days of vexation from fever and physic, with his strength but little impaired by depletion—it secures him from the hazard of a malignant paroxysm—from the doubtful issue of the typhoid state—doubtful under any of the many modes of treatment; and it will never impose upon the physician the fearful alternative of allowing the disease to run its course towards a doubtful issue, or to adopt a heroic course of mercury, which may end in salivation—an artificial disease, infinitely more annoying and of longer duration than the one it may have substituted—which may at last end in the loss of the patient's teeth, or of his lips, or of his life. Fearful indeed is the choice of the latter alternative; and far better, that the profession should lay under the reproach of impotency to save human life, than the more terrible one of sacrificing it.

When it is remembered that remittent fever is the endemic disease of Southern climates, the necessary exposure of the population in the summer and autumn, and the universality of its attacks, and the high rates of its mortality, under every mode of treatment hitherto adopted, and if the success of the abortive method has been here truly represented; then it may not be deemed extravagant to say—that its universal adoption throughout the Southern country, would confer blessings, within that sphere, proportionate to those conferred upon the world by the discovery of vaccination. It is gratifying to know, that it is fast winning its way to universal adoption; and the claim to the honor of diffusing the knowledge of this treatment, in this region of the Southern country, set forth in behalf of the Medical College of Georgia, by Professor Dugar, in his recent introductory lecture, is unhesitatingly endorsed by the writer. Here, the principle of this method was distinctly and publicly announced, as early as 1836, and ever since, its alumni, fully indoctrinated in the principles of this method, scattered through this and the neighboring States, have freely used the influence, which their unprecedented success in the treatment of bilious fever, has secured to them, in extending the same principles far and wide among their brethren of the faculty. It wins its way readily to the willing and candid enquirer, and *compels* the assent of the reluctant.

2. *To moderate the violence of the paroxysm.*—If the congestions and inflammations manifested with increased violence during the paroxysms are accidents, they do yet materially affect the issue of the case, and must command attention. But it is not the intention of the writer, at present, to enlarge upon this part of the treatment, the circumstances under which the various means of the antiphlogistic method may or may not be used, having been so judiciously defined in the works on

Practice. It was his intention to have added cases, to show how fairly the principles of pathology and practice, here advocated, are deduced from facts; but circumstances forbid the extension of this article.

